# Systematic Review of the Application of Pulmonary Hypertension Treatments in Ventricular Septal Defect, Pulmonary Atresia, and Major Aortopulmonary Collateral Arteries

**DOI:** 10.3390/jcm15031087

**Published:** 2026-01-30

**Authors:** Keiichi Hirono, Keiko Uchida, Taku Ishii, Hidekazu Ishida, Shinichi Takatsuki, Hiroyuki Fukushima, Kei Inai, Susumu Hosokawa, Reina Ishizaki, Hirofumi Sawada, Naofumi F. Sumitomo, Ayako Chida-Nagai, Yuichi Ishikawa, Hirohiko Motoki, Atsushi Yao, Shigetoyo Kogaki, Hiroyuki Yamagishi, Shozaburo Doi

**Affiliations:** 1 Department of Pediatrics, Toyama University Hospital, Toyama 930-0194, Japan; 2 Department of Physiology, Tokyo Medical University, Tokyo 160-8402, Japan; 3 Department of Pediatrics, Faculty of Medicine, Institute of Science Tokyo, Tokyo 113-8510, Japan; 4 Department of Pediatrics, Osaka University Graduate School of Medicine, Suita 565-0871, Japan; 5 Department of Pediatrics, Toho University, Omori Medical Center, Tokyo 143-8541, Japan; 6 Department of Pediatrics, Tokyo Dental College Ichikawa General Hospital, Ichikawa 272-8513, Japan; 7 Department of Pediatric Cardiology and Adult Congenital Cardiology, Tokyo Women’s Medical University, Tokyo 272-8513, Japan; 8 Department of Pediatrics, Japanese Red Cross Musashino Hospital, Musashino 180-8610, Japan; 9 Department of Pediatrics, School of Medicine, Keio University, Tokyo 160-8582, Japan; 10 Department of Pediatrics, Graduate School of Medicine, Mie University, Tsu 514-8507, Japan; 11 Department of Pediatrics, Hokkaido University Hospital, Sapporo 060-8648, Japan; 12 Minatomirai Medical Corporation, Chigasaki Kanazawa Internal Medical Clinic, Chigasaki 253-0052, Japan; 13 Department of Cardiology, Shinshu University, Matsumoto 390-8621, Japan; 14 Health Service Promotion, The University of Tokyo, Tokyo 113-8654, Japan; 15 Department of Pediatrics, Osaka General Medical Center, Osaka 558-8558, Japan; 16 Department of Pediatrics, Tokyo Metropolitan Children’s Medical Center, Tokyo 183-8561, Japan

**Keywords:** pulmonary atresia, ventricular septal defect, major aortopulmonary collateral artery, pulmonary hypertension

## Abstract

**Background:** Pulmonary atresia (PA) with ventricular septal defect (VSD) and major aortopulmonary collateral arteries (MAPCAs), a life-threatening congenital heart defect (CHD), is frequently associated with abnormal pulmonary blood flow and vascular remodeling, causing hypoxia and heart failure. Segmental pulmonary hypertension (PH), a distinct PH type, may exist in some patients. Pulmonary vasodilators have been considered for treatment; however, evidence of their efficacy and safety remains lacking. **Methods:** A systematic review was conducted using PubMed, MEDLINE, The Cochrane Library, and Ichushi Web, encompassing studies from inception to May 2023. Inclusion criteria focused on patients with PA/VSD/MAPCAs treated with PH medications. **Results:** Of 86 studies screened, 6 met the inclusion criteria, including 1 cohort study and 5 case reports, comprising 22 patients. The most frequently administered medications were sildenafil (14 cases) and bosentan (12 cases), with 16 patients receiving monotherapy. Clinical improvements were observed in pulmonary vascular resistance (8/8 patients), oxygen saturation (8/19 patients), and symptoms (19/21 patients). Adverse effects were noted in five patients, including treatment discontinuation in two. **Conclusions:** PH medications may benefit some patients with PA/VSD/MAPCAs; however, the extremely limited sample size (*n* = 22) and substantial heterogeneity in anatomy, age, surgical status, and treatment regimens severely limit interpretability and clinical applicability. Considering the potential benefits and risks associated with these medications, their use should be considered cautiously and restricted to specialized centers with expertise in CHD and PH management.

## 1. Introduction

Pulmonary atresia (PA) with ventricular septal defect (VSD) and major aortopulmonary collateral arteries (MAPCAs) represents one of the most complex and severe congenital heart diseases, occurring in approximately 0.07 per 1000 live births, constituting approximately 2.5% of all congenital heart defects (CHDs) [1]. The anatomical complexity of these cases, characterized by absent or severely hypoplastic central pulmonary arteries and the presence of MAPCAs to supply pulmonary blood flow, poses significant challenges for both diagnosis and management [2,3,4]. MAPCAs embryologically originate from somatic arterial systems during fetal development, contributing to their diverse presentations. This heterogeneity frequently causes pulmonary vasculature segments to experience high or low pulmonary blood flow, contributing to variable responses and complicating therapeutic approaches.

The therapeutic strategy for cases of PA/VSD/MAPCA typically encompasses palliative surgical interventions, including shunt placement, or more definitive procedures, including unifocalization of the pulmonary arteries or complete intracardiac repair (Rastelli procedure). However, some patients remain at risk for developing persistent pulmonary hypertension (PH), specifically segmental PH, primarily due to residual or progressive vascular remodeling, which is categorized as Group 5.4 PH associated with CHD [5,6]. PAH-targeted therapies, including selective pulmonary vasodilators, may hold therapeutic potential for these patients [7]; however, a critical gap exists in the literature regarding the application of these therapies in cases of PA/VSD/MAPCA, with limited data predominantly derived from case reports and small cohort studies. Therefore, the role of these medications remains unclear, and their routine use has not been standardized, warranting further investigation.

Contemporary surgical outcome studies have documented substantial improvements in survival for patients with pulmonary atresia with ventricular septal defect (PA/VSD) and MAPCAs, with reported 10-year survival >80% in cohorts with effective staged or unifocalization repair, although non-confluent pulmonary arteries remain a strong mortality predictor [8].

Nevertheless, a subset of patients develop persistent or progressive pulmonary vascular disease and pulmonary hypertension post-repair, and scattered case reports have described the use of pulmonary vasodilators like sildenafil demonstrating symptomatic improvements in individual cases [9].

Segmental pulmonary hypertension associated with PA/VSD/MAPCAs represents a heterogeneous vascular phenotype that complicates physiologic response to vasodilator therapy and highlights the necessity for structured evidence synthesis [10].

This systematic review aimed to evaluate the current evidence surrounding the application of PH medications in patients with PA/VSD/MAPCA, assessing both therapeutic outcomes, including reductions in pulmonary vascular resistance (PVR) and improvements in oxygen saturation, and the associated risks, including adverse events and treatment discontinuation. Furthermore, this review sought to determine whether these treatments should be recommended for use in specialized centers experienced in CHD and PH management.

## 2. Materials and Methods

This systematic review was conducted following the PRISMA 2020 guidelines (https://www.prisma-statement.org/prisma-2020, accessed on 27 January 2026) (Table S1). This systematic review was not registered in PROSPERO or any other registry. The purpose was to evaluate the efficacy of PH treatment in patients with PA/VSD/MAPCA-associated complex CHD.

### 2.1. Eligibility Criteria

Inclusion criteria encompassed studies involving patients with PA/VSD/MAPCA who underwent treatment for PH. We included both cohort studies and case reports that provided clear documentation of patient outcomes following PH medication administration. The search results did not reveal randomized controlled trials (RCTs) and case–control studies. No age and medication restrictions were imposed.

### 2.2. Information Sources

A comprehensive literature search was performed using the following databases: PubMed, MEDLINE, The Cochrane Library, and Ichushi Web (a Japanese medical database). The search was conducted from inception up to May 2023. These databases were selected to ensure broad coverage of international and Japan-based studies on PH in congenital heart disease.

### 2.3. Search Strategy

To extract relevant studies, the search terms included “MAPCA,” “VSD,” “PA,” and “PH,” combined with “treatment” or “therapy” (Table S2). To refine the search, Boolean operators were used, and filters were applied to limit the results to original research articles in English.

### 2.4. Selection Process

The selection process comprised a two-step screening procedure. First, two reviewers independently screened titles and abstracts. Discrepancies were resolved through discussion or consultation with a third reviewer. Full-text articles of selected abstracts were retrieved and evaluated against the inclusion criteria. Only studies that met all criteria were included in the final analysis.

### 2.5. Data Collection Process

Using a standardized data extraction form, two reviewers independently extracted data from each included study. The data extracted included patient demographics, details of the PH treatments administered, and the reported outcomes (e.g., symptoms, pulmonary artery pressure [PAP], and oxygen saturation [SpO_2_]). Any inconsistencies in the data extraction were resolved through discussion.

### 2.6. Data Items

The changes in clinical symptoms, PVR, PAP, and SpO_2_ were the primary outcomes of interest. Reported adverse effects of the medications and mortality rates encompassed the secondary outcomes. Moreover, data on the type of surgery performed (if any) and the timing of PH medication administration relative to surgical interventions were collected.

### 2.7. Risk of Bias Assessment

The Cochrane Risk of Bias Tool for observational studies was used for assessing the risk of bias of the included studies. Studies were examined for selection, measurement, and reporting biases.

### 2.8. Synthesis Methods

Owing to the limited number of included studies and the heterogeneity of the patient populations and interventions, a meta-analysis was not performed. Instead, a qualitative synthesis was conducted. To identify common trends and notable discrepancies in the outcomes reported, the findings were summarized and compared across studies.

### 2.9. Certainty of Evidence

The quality of evidence for each outcome was evaluated using the GRADE approach, which considers factors, including study design, risk of bias, consistency of results, and directness of evidence.

## 3. Results

### 3.1. Study Selection

**Figure 1:**
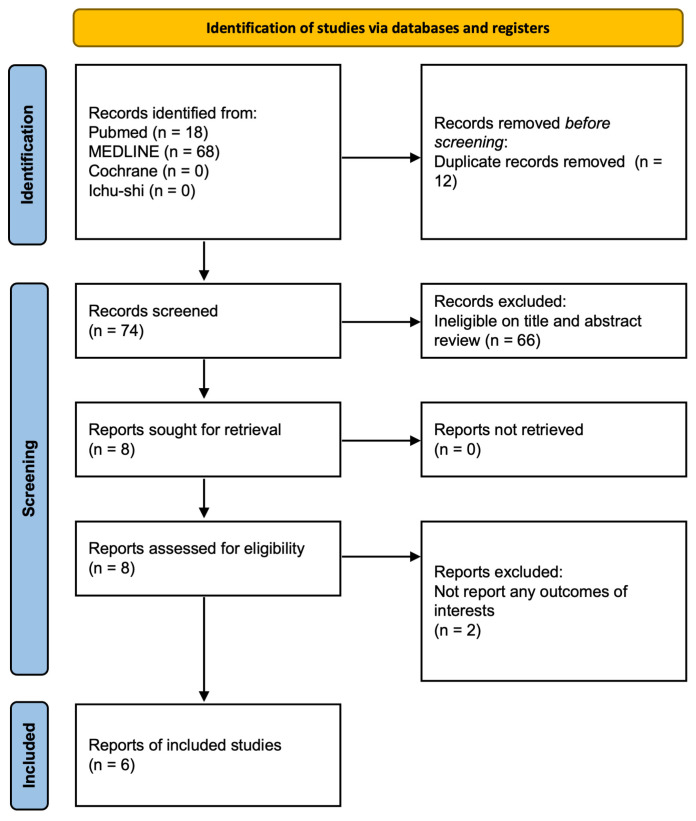
PRISMA flow diagram of study screening and selection.

Overall, 86 studies were extracted through the initial database searches using PubMed, MEDLINE, The Cochrane Library, and Ichushi Web (Figure 1). After reviewing the titles and abstracts, eight studies were selected for full-text review. Subsequently, six studies were included in the final synthesis, comprising one cohort study and five case reports (Table 1) [9,11,12,13,14,15]. These studies mainly focused on the use of PH drugs in patients with MAPCA-associated complex CHDs, specifically PA/VSD/MAPCA.

### 3.2. Study Characteristics

The six included studies investigated a total of 22 patients (Table 1). Of these patients, 16 (73%) involved single-drug therapy, whereas six (27%) involved combination therapy for PH treatment. The drugs administered encompassed sildenafil, bosentan, selexipag, ambrisentan, intravenous prostacyclin, and inhaled treprostinil in 14, 14, 2, 1, 1, and 2 patients, respectively. The following were the surgical interventions performed at the time of administering PH treatment for PA/VSD/MAPCA: three patients without surgery, three patients with Blalock–Taussig shunt surgery, two patients with unifocalization, one patient with Brock procedure, six patients with right ventricle–pulmonary artery (RV–PA) conduit only, and seven patients with RV–PA conduit and VSD closure. Surgical palliation preceded pulmonary vasodilator therapy initiation in 13 of 22 patients (59%). The median interval between the most recent surgical intervention and drug initiation was 3.4 (interquartile range, 0.6–9.8; range, 0.1–33) years. The remaining nine patients (41%) received medical therapy without prior surgery.

### 3.3. Clinical Outcomes

**Figure 2:**
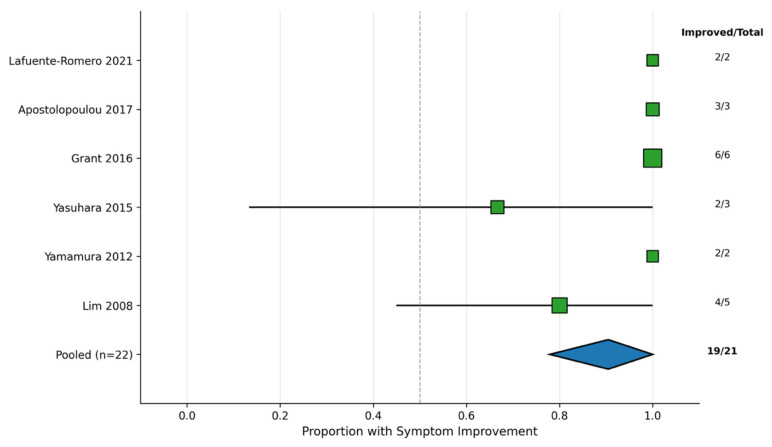
Forest plot of symptom improvement rates by study. Square sizes are proportional to sample sizes. Error bars represent 95% confidence intervals calculated using the Wald method. The diamond represents the descriptive pooled estimate across all studies (19/21 patients improved, 90%) [9,11,12,13,14,15].

Outcomes were reported across 21 patients, including 19 patients (90%) demonstrating improvement in clinical symptoms and two patients exhibiting deterioration (Table 1 and Table 2; Figure 2). Symptom improvement was assessed using heterogeneous methods across studies. Among 21 patients with documented symptom assessment, 19 (90%) demonstrated improvement, whereas two patients exhibited deterioration. Specifically, functional class improvement (≥1 NYHA class) was observed in 8 of 10 patients where this outcome was reported. Six-minute walk test distance improved in 5 of 6 patients who underwent this assessment, with increases ranging from 30 to 160 m. Subjective improvement in exercise tolerance, reduced dyspnea, and enhanced ability to perform daily activities were reported in 16 of 18 patients with available data. Three patients were able to discontinue supplemental oxygen therapy following treatment initiation. SpO_2_ levels were reported in 19 patients, with baseline values showing a median of 81% (range: 52–97%). Improvements were observed in eight patients with a median increase of 10 percentage points (range: +3 to +26), whereas 11 patients showed no improvement (range: −10 to +2 percentage points). Post-treatment SpO_2_ showed a median of 84% (range: 68–98%). PVR index (PVRI) was reported in eight patients, with baseline PVRI showing a median of 6.55 Wood units × m^2^ (range: 3.1–17.1). All eight patients exhibited improvement, with post-treatment PVRI showing a median of 2.45 Wood units × m^2^ (range: 2.2–6.8), representing a median reduction of 43% (range: 29–51%). PAP was reported in nine patients, with seven patients exhibiting improvements, whereas two patients demonstrated no improvement. Cardiac index (CI) was reported in eight patients, with three patients showing improvements, whereas five patients exhibited no improvement. Symptomatic improvement was comparable between patients who underwent previous surgery (11/13, 85%) and those without surgery (7/9, 78%). All four deaths occurred within 2 years of therapy, two following earlier palliative surgery and two that occurred in surgically naïve patients.

### 3.4. Mortality and Adverse Events

Of the 22 patients, four deaths (18%) were reported, with three occurring in patients receiving sildenafil and one in a patient receiving bosentan (Table 1). Two deaths occurred in young children (2.2-year-old female and 2.3-year-old male), one in a young adult (19-year-old female), and one in a middle-aged adult (47-year-old male). Three patients received sildenafil monotherapy, and one received bosentan monotherapy. The follow-up periods ranged from 0.6 to 1.4 years.

Importantly, none of the reported deaths were directly attributed to PH medication adverse effects in the original studies. Grant et al. explicitly reported that both deaths in their cohort (2.2-year-old and 2.3-year-old) were unrelated to PH medications; one death occurred following elective RV–PA conduit replacement complicated by postoperative hemorrhage requiring extracorporeal membrane oxygenation, and the other resulted from persistent hypoxia associated with extremely hypoplastic pulmonary arteries. In the study by Lim et al., the 19-year-old female with 22q11.2 deletion syndrome died from progressive heart failure despite intensive medical management, and the 47-year-old male died from pneumonia complicated by hemoptysis, respiratory failure, and multi-organ failure seven months after initiating therapy. These findings suggest that mortality in this population is primarily driven by the inherent severity and complexity of the underlying cardiac disease, postoperative complications, or intercurrent illness rather than direct PH medication toxicity. Nevertheless, the high mortality rate in young patients highlights the need for careful patient selection and close monitoring when initiating PH therapy in this vulnerable population.

Notably, one deceased patient was a 19-year-old female with 22q11.2 deletion syndrome who underwent palliative RV–PA conduit surgery and received sildenafil treatment for 1 year. Although the exact causes of death are unclear, the high mortality rate in young patients (aged 2–3 years) receiving sildenafil warrants careful consideration, and the underlying disease severity appears to be a more significant contributor to outcomes than the medication itself. Adverse effects were reported in five cases (23%), with mild side effects in three cases (sildenafil, bosentan, and treprostinil) and more severe effects resulting in drug discontinuation in two cases (sildenafil and bosentan).

### 3.5. Risk of Bias and Evidence Quality

The included studies were mainly case reports and a single cohort study, limiting the overall quality and generalizability of the evidence. The risk of bias was assessed as moderate to high owing to the small sample sizes and the lack of RCTs (Figure S1). Consequently, the evidence supporting the use of PH drugs in patients with PA/VSD/MAPCA was considered to be of very low quality.

### 3.6. Heterogeneity and Interpretability Limitations

The interpretability of the synthesized evidence is severely constrained by multiple sources of heterogeneity (Table 2). First, the anatomical heterogeneity inherent to PA/VSD/MAPCA—including variable MAPCA size, number, origin, stenosis severity, and pulmonary vascular bed development—precludes direct comparison of treatment responses across patients. Second, the wide age range (0.5–47 years) encompasses distinct physiological states, from rapidly growing infants with evolving pulmonary vasculature to adults with established pulmonary vascular disease, each potentially responding differently to vasodilator therapy. Third, the surgical status at treatment initiation varied substantially: three patients had no prior intervention, three had only Blalock–Taussig shunts, and seven had undergone complete repair with VSD closure, representing fundamentally different hemodynamic substrates. Fourth, treatment regimens lacked standardization, with variations in medication class, dosing protocols, and duration of therapy. Finally, outcome assessment methods differed across studies, with inconsistent reporting of hemodynamic parameters, exercise capacity, and adverse events.

These limitations have direct implications for clinical applicability. The current evidence does not permit identification of patient subgroups most likely to benefit from PH therapy, optimal timing of treatment initiation relative to surgical intervention, preferred medication or combination regimens, or appropriate monitoring protocols. Clinicians should interpret the reported improvement rates with extreme caution, recognizing that the observed responses may not be reproducible across the heterogeneous spectrum of patients with PA/VSD/MAPCA.

## 4. Discussion

This systematic review aimed to assess the efficacy and safety of PH treatments in patients with PA/VSD/MAPCA, a rare and life-threatening condition characterized by abnormal pulmonary vasculature development and blood flow dynamics. The results suggest that selective pulmonary vasodilators have demonstrated positive outcomes in some cases, whereas the overall quality of evidence remains weak owing to the complexity of the disease and the heterogeneity of available studies.

### 4.1. Relationship Between MAPCA Anatomy, Hemodynamics, and Treatment Response

The heterogeneous response to pulmonary hypertension-targeted therapies observed in this review appears closely linked to MAPCA anatomy and its associated hemodynamic profiles. Patients with relatively confluent pulmonary arteries or segmental pulmonary hypertension, in whom pulmonary blood flow is distributed through discrete vascular territories with elevated but potentially reversible pulmonary vascular resistance, were more likely to demonstrate symptomatic or hemodynamic improvement following vasodilator therapy. In contrast, patients with severely hypoplastic native pulmonary arteries, extensive abnormal MAPCA arborization, or dominant low-resistance collateral vessels supplying large pulmonary segments showed less consistent benefit. In these anatomical settings, pulmonary vasodilators may preferentially increase flow to already well-perfused segments, exacerbating pulmonary overcirculation and ventricular volume overload rather than reducing overall pulmonary vascular resistance. Furthermore, marked intersegmental differences in pulmonary vascular resistance—a hallmark of MAPCA-dependent circulation—may limit uniform vasodilatory effects and contribute to unpredictable responses. These hemodynamic characteristics provide a plausible explanation for the observed variability in treatment response, including non-response and clinical deterioration in some patients. Together, these observations underscore that MAPCA anatomical subtype and baseline hemodynamic assessment are central determinants of response to PH therapy and should be integral to patient selection and therapeutic decision-making in PA/VSD/MAPCAs.

### 4.2. Application of PH Treatments in Patients with PA/VSD/MAPCA

The Sixth World Symposium on Pulmonary Hypertension, held in 2018 in Nice, classified complex CHD as under Group 5.4, “PH with unclear and/or multifactorial mechanisms,” within Group 5, indicating PH of unknown or multifactorial origin [5,6]. PA/VSD/MAPCA falls under this category of segmental PH in Group 5.4, which is a pulmonary vascular disease occurring in one or more pulmonary segments. Patients with PA/VSD/MAPCA frequently present significant challenges owing to the anatomical and physiological variability in their pulmonary vasculature, as outlined in previous studies [16]. In cases of PA/VSD/MAPCA, the MAPCAs are characterized by hypoplastic or unevenly developed pulmonary arterial beds, peripheral pulmonary artery stenosis, and vasospasm in poorly ventilated alveolar regions, with potential involvement of pulmonary vascular remodeling [17]. Segmental PH may arise owing to increased pulmonary blood flow through nonstenotic collateral arteries or systemic-to-pulmonary shunts, hypoplastic or unevenly developed pulmonary arterial beds, vasospasm, and peripheral pulmonary artery stenosis [14]. In PA/VSD/MAPCA, the hypoplastic or segmentally uneven pulmonary arterial beds are susceptible to vascular remodeling, even under low pulmonary blood flow conditions. Interestingly, a histological analysis of the collateral arteries in patients with segmental PH revealed medial thickening and intimal proliferation, similar to the vascular remodeling observed in idiopathic pulmonary arterial hypertension (IPAH) [7].

Patients with PA/VSD/MAPCA, particularly those without PH, who undergo complete repair surgery exhibit favorable long-term outcomes and have a good prognosis. When surgery is performed during infancy, the 3- to 10-year survival rate exceeds 85%, and the 15-year survival rate reaches 78% [18]. Conversely, studies have indicated that the average age of death in patients who reach adulthood without surgery ranges 20–40 years, and segmental PH negatively influences their prognosis [19,20]. The present synthesis suggests that the chronology of surgical palliation and pharmacotherapy did not significantly affect short-term symptomatic response; however, mortalities were observed irrespective of surgical status. Considering that pulmonary vasodilators are increasingly administered as a “bridge” to delayed complete repair, our findings highlight the significance of prospective studies evaluating optimal sequencing and timing. The mortality-related data obtained in this review emphasize crucial safety considerations for PH treatment in patients with PA/VSD/MAPCA. The high mortality rate in young children (aged 2–3 years) and the involvement of sildenafil in three of four fatal cases suggest that age-specific dosing protocols and improved monitoring are required. Although direct causality between PH medications and mortality cannot be established from the available data, the underlying disease complexity and hemodynamic instability in these patients may contribute to poor outcomes.

This review identified several cases wherein pulmonary vasodilators, including sildenafil and bosentan, caused improvements in clinical parameters, including PVR and SpO_2_. These results align with the hypothesis that PH treatments developed for IPAH may benefit patients with PA/VSD/MAPCA; however, the therapeutic response was not uniform across all patients. Some patients developed adverse effects, including drug intolerance and worsening symptoms, highlighting the significance of careful patient selection and tailored treatment approaches. For instance, MAPCAs can present with variable sizes and degrees of stenosis, complicating the overall response to PH medications. As previously discussed, some MAPCAs may be vulnerable to progressive narrowing or occlusion, whereas others may contribute to over-perfusion, exacerbating the clinical picture with high pulmonary blood flow and subsequent heart failure. The diversity of MAPCAs and the differences in surgical techniques and timing for each patient contribute to the variations in the effectiveness of PH treatment in patients with PA/VSD/MAPCA. Furthermore, this diversity poses difficulties in accumulating cases and conducting data analysis, complicating the development of strategies for PH treatment in patients with PA/VSD/MAPCA.

### 4.3. Analysis of Fatal Cases and Prognostic Considerations

The detailed analysis of fatal cases in this review provides important insights into the clinical application of pulmonary vasodilator therapy in PA/VSD/MAPCAs. Several key observations emerge. First, the presence of genetic syndromes, particularly 22q11.2 deletion, may be associated with more severe pulmonary vascular abnormalities and poorer outcomes. Among the four fatal cases summarized in Table 1, one fatal patient reported by Lim et al. (Table 1) had documented 22q11.2 deletion syndrome, and two patients in the non-fatal cohort reported by Grant et al. (Table 1) also carried this genetic abnormality. The association between 22q11.2 deletion and hypoplastic pulmonary arterial trees has been previously reported and may represent an important prognostic factor. Second, the severity of pulmonary artery hypoplasia and abnormal MAPCA arborization appears to be a critical determinant of treatment response. Patients with marked arborization abnormalities, such as the fatal patient reported by Lim et al. (Table 1), or extremely hypoplastic pulmonary arteries, such as the fatal patient reported by Grant et al. (Table 1), showed limited sustained benefit despite initial symptomatic improvement, suggesting that the anatomical substrate may ultimately limit the efficacy of pulmonary vasodilator therapy. Third, pre-existing ventricular dysfunction, particularly biventricular involvement, may identify patients at higher risk for treatment failure. In the fatal patient reported by Lim et al. (Table 1), severe right ventricular dysfunction accompanied by moderate left ventricular dysfunction was documented at baseline, and progressive heart failure was the ultimate cause of death despite pulmonary vasodilator therapy. Fourth, advanced age and multiple comorbidities in adult patients may further increase vulnerability to adverse outcomes. For example, the adult fatal patient reported by Grant et al. (Table 1) demonstrated marked symptomatic improvement with therapy but later died from an infectious complication unrelated to pulmonary hypertension progression. Taken together, these observations indicate that patient selection is critical when considering pulmonary vasodilator therapy in PA/VSD/MAPCAs. Patients with severely hypoplastic pulmonary arterial beds, pronounced arborization defects, significant ventricular dysfunction, or multiple systemic comorbidities may derive less sustained benefit and therefore require particularly careful monitoring. Future studies should aim to identify biomarkers or imaging parameters capable of predicting treatment response and guiding therapeutic decision-making in this heterogeneous population.

### 4.4. Non-Responders and Clinical Deterioration

While the majority of reported patients demonstrated symptomatic or hemodynamic improvement, a subset of patients did not benefit from pulmonary hypertension-targeted therapy or experienced clinical deterioration, including death. Understanding the characteristics of these non-responders is critical for interpreting the potential limitations and risks of PH therapy in PA/VSD/MAPCA. Several pathophysiological mechanisms may account for the lack of benefit or adverse outcomes observed in these patients. Advanced pulmonary vascular remodeling, characterized by irreversible intimal fibrosis and loss of vasoreactivity, may render pulmonary vasodilators ineffective. In such cases, reductions in pulmonary vascular resistance are unlikely, and disease progression may continue despite therapy. In addition, unfavorable or heterogeneous pulmonary hemodynamics, a hallmark of PA/VSD/MAPCA, may contribute to poor response. Patients with severely hypoplastic pulmonary arteries, abnormal MAPCA arborization, or marked segmental differences in pulmonary vascular resistance may not experience uniform vasodilation. Instead, pulmonary vasodilators may preferentially increase blood flow to low-resistance segments, potentially exacerbating pulmonary overcirculation, ventricular volume overload, or heart failure. Furthermore, advanced ventricular dysfunction or longstanding cyanosis may limit the capacity to tolerate increased pulmonary blood flow, particularly in patients with incomplete surgical repair or persistent systemic-to-pulmonary shunting. These factors may partially explain why clinical deterioration and mortality were observed within a relatively short period after therapy initiation in some cases. Collectively, these findings underscore that PH therapy is not universally beneficial in PA/VSD/MAPCA and that careful patient selection based on detailed anatomical, hemodynamic, and ventricular assessment is essential. The identification of predictors of non-response remains an important unmet need and highlights the necessity for prospective studies and multicenter registries.

### 4.5. Causal Relationship Between PH Therapy and Mortality

Although pulmonary hypertension-targeted therapies were associated with improvements in symptoms and hemodynamic parameters in a subset of patients, the overall mortality rate observed in this systematic review was 18%. Importantly, this finding should not be interpreted as evidence of treatment-related harm or benefit, as the available data are derived from heterogeneous case reports and a single small cohort study, precluding any assessment of causality. Mortality in this population is multifactorial and likely reflects the intrinsic severity of PA/VSD/MAPCA, advanced pulmonary vascular disease, ventricular dysfunction, and limited surgical options rather than the direct effects of PH medications. Importantly, there is also a theoretical concern that PH-targeted therapies may exacerbate pulmonary overcirculation or heart failure in selected anatomical settings. In patients with PA/VSD/MAPCA, pulmonary blood flow is often supplied by MAPCAs with highly variable resistance and arborization. Non-selective pulmonary vasodilation may preferentially increase blood flow to already well-perfused or low-resistance segments, potentially resulting in pulmonary overcirculation, volume overload, or worsening ventricular function, particularly in patients with incomplete unifocalization or residual systemic-to-pulmonary shunts. Such mechanisms may be especially relevant in cases with heterogeneous segmental pulmonary hypertension, where vasodilator therapy could theoretically aggravate hemodynamic imbalance rather than uniformly reducing pulmonary vascular resistance. Although these risks remain speculative and were not systematically evaluated in the included studies, they underscore the need for careful patient selection, detailed anatomical and hemodynamic assessment, and close monitoring when considering PH therapy in this complex population. Therefore, while PH medications may offer symptomatic or hemodynamic benefit in selected patients with PA/VSD/MAPCA, their use should be approached with caution and restricted to specialized centers with expertise in congenital heart disease and pulmonary hypertension, ideally within a multidisciplinary framework that integrates surgical, interventional, and medical strategies.

### 4.6. Limitations of the Evidence

This review revealed that the evidence base for PH treatment in patients with PA/VSD/MAPCA is limited and of low quality. Most included studies were case reports and small cohort studies, and RCTs were lacking. The heterogeneity of the patient population in terms of MAPCA anatomy, pulmonary artery development, and hemodynamic status further complicates the generalizability of the findings. Moreover, long-term follow-up data are scarce, making evaluating the sustained efficacy and safety of PH treatments in these patients challenging.

The absence of standardized treatment protocols for patients with PA/VSD/MAPCA represents another limitation. Currently, no consensus on the optimal timing and combination of PH medications as well as on the selection of patients who would benefit most from these treatments is available. As noted, the progression of pulmonary vascular disease and the potential for obstructive lesions in MAPCAs, which vary across patients, add complexity to treatment decisions.

### 4.7. Limitations of the Review Process

The limited number of available studies and the exclusion of non-English publications, which may have introduced selection bias, also constrained this review. Additionally, the search strategy may have missed relevant unpublished data or studies not indexed in the databases consulted. Furthermore, this review did not consider the costs or long-term resource implications of the treatments investigated. Lastly, the absence of RCTs limits the strength of the conclusions, as the current evidence is primarily drawn from observational studies with inherent biases.

### 4.8. Implications for Practice, Policy, and Future Research

The results of this review suggest that PH treatment in patients with PA/VSD/MAPCA should be cautiously considered and primarily applied in specialized centers with expertise in CHD and PH management. The potential benefits of these treatments, particularly PVR and SpO_2_ improvement, should be balanced against the risk of adverse effects, including pulmonary vascular disease progression and drug-related complications. Considering the limited evidence, treatment decisions should be tailored on the basis of each patient’s specific hemodynamic profile and anatomical characteristics.

Given the rarity and anatomical heterogeneity of PA/VSD/MAPCA, establishing robust evidence for pulmonary hypertension-targeted therapies remains challenging. To advance the field, several concrete research directions should be prioritized. First, the creation of international, multicenter registries dedicated to PA/VSD/MAPCA-associated pulmonary hypertension is essential. Such registries would enable the systematic collection of longitudinal data across diverse anatomical subtypes, surgical histories, and treatment strategies, thereby improving statistical power and external validity. Second, future studies should adopt standardized reporting of hemodynamic parameters and clinical outcomes, including pulmonary vascular resistance, segmental pulmonary artery pressures, cardiac index, oxygen saturation, functional class, and adverse events. Uniform reporting would facilitate cross-study comparisons and allow more meaningful synthesis of observational data. Third, given that many patients receive PH therapy during childhood, age-specific safety and efficacy analyses are critically important. Developmental differences in pulmonary vasculature, drug metabolism, and long-term exposure necessitate stratified analyses across pediatric and adult populations to better define risk–benefit profiles. Finally, because randomized controlled trials are unlikely to be feasible in this rare and heterogeneous population, pragmatic, well-designed observational studies, including prospective cohorts and registry-based analyses, should be pursued. When combined with careful phenotyping and standardized outcome measures, such approaches may provide the most realistic pathway to evidence generation and clinical guidance.

## 5. Conclusions

This systematic review demonstrates that PH medications exhibit potential benefits in patients with PA/VSD/MAPCA, with improvements observed in PVR, symptoms, and oxygen saturation in selected cases. However, the evidence remains limited to small cohort studies and case reports, indicating significant mortality and adverse effects. Considering the complexity of this patient population and the lack of RCTs, PH medications should be cautiously administered and only in specialized centers with expertise in CHD and PH management. Treatment decisions should be individualized on the basis of surgical status, hemodynamic profile, and patient-specific factors. Future research priorities encompass registry-based studies and RCTs to develop evidence-based guidelines for this vulnerable population. In conclusion, the application of pulmonary vasodilators in this population is weakly recommended, with a strong emphasis on specialist consultation and management in experienced centers.

## Figures and Tables

**Table 1:** Study details and patient demographics.

Authors, Year	Age (Year)	Sex	Chromosome	Follow-Up Period (Years)	Surgical Interventions Before PH Treatment	PH Treatment	Improvement in Cardiac Parameters						Improvement in Clinical Symptoms	Adverse Events	Outcome
							SpO_2_	PVR	PAP	CI	NYHA	6MWD			
Lafuente-Romero, A., 2021 [11]	12	male	N/A	3.3	Rastelli procedure	sildenafil, bosentan, and selexipag	N/A	N/A	N/A	N/A	improved	improved	yes	yes; mild	alive
	6	female	N/A	1.2	UF	sildenafil, bosentan, and selexipag	not changed	N/A	N/A	N/A	improved	improved	yes	yes; mild	alive
Apostolopoulou, S.C., 2017 [12]	26	female	N/A	14	no	bosentan	not changed	N/A	N/A	N/A	improved	not changed	yes	N/A	alive
	21	male	N/A	10	BT shunt	bosentan	not changed	N/A	N/A	N/A	improved	not changed	yes	N/A	alive
	31	female	N/A	5	BT shunt	bosentan	improved	N/A	N/A	N/A	improved	N/A	yes	N/A	alive
Grant, E.K., 2016 [13]	1.3	female	no	4.7	Rastelli procedure	sildenafil, bosentan, and treprostinil (INH)	improved	improved	not changed	improved	N/A	N/A	yes	yes; mild	alive
	2.2	female	no	0.8	palliative RV–PA connection	bosentan	improved	improved	improved	not changed	N/A	N/A	no	N/A	dead
	2.3	male	no	1.4	no	sildenafil	improved	improved	deteriorated	not changed	N/A	N/A	N/A	N/A	dead
	4	male	22q11.2	2	UF	sildenafil	not changed	improved	improved	deteriorated	N/A	N/A	yes	N/A	alive
	4	male	no	3	no	sildenafil and bosentan	deteriorated	improved	improved	deteriorated	N/A	N/A	yes	N/A	alive
	4	male	no	17	Rastelli procedure	sildenafil, bosentan, prostacyclin (IV), ambrisentan, and treprostinil (INH)	improved	N/A	N/A	N/A	N/A	N/A	yes	N/A	alive
	5	male	no	6	palliative RV–PA connection	sildenafil	improved	improved	improved	not changed	N/A	N/A	yes	N/A	alive
Yasuhara, J., 2015 [14]	5	female	22q11.2	0.3	Rastelli procedure	sildenafil and bosentan	improved	N/A	N/A	N/A	N/A	N/A	yes	no	alive
	18	male	22q11.2	5	BT shunt	bosentan	not changed	N/A	N/A	N/A	N/A	N/A	yes	no	alive
	32	male	no	0.1	Rastelli procedure	bosentan	deteriorated	N/A	N/A	N/A	N/A	N/A	no	yes; severe	alive
Yamamura, K., 2012 [15]	5	female	N/A	2	Rastelli procedure	bosentan	N/A	improved	improved	improved	N/A	N/A	yes	no	alive
	0	male	N/A	3	Rastelli procedure	bosentan	N/A	improved	improved	improved	N/A	N/A	N/A	no	alive
Lim, Z.S., 2008 [9]	19	female	22q11.2	1	palliative RV–PA connection	sildenafil	not changed	N/A	N/A	N/A	N/A	N/A	yes	N/A	dead
	47	male	no	0.6	Brock	sildenafil	not changed	N/A	N/A	N/A	N/A	improved	yes	N/A	dead
	18	female	no	N/A	palliative RV–PA connection	sildenafil	not changed	N/A	improved	N/A	N/A	N/A	yes	yes; severe	alive
	17	female	no	1	palliative RV–PA connection	sildenafil	not changed	N/A	N/A	N/A	N/A	N/A	yes	N/A	alive
	38	female	22q11.2	0.3	palliative RV–PA connection	sildenafil	improved	N/A	N/A	N/A	N/A	improved	yes	N/A	alive

SpO_2_, saturation of percutaneous oxygen; PVR, pulmonary vascular resistance; PAP, pulmonary arterial pressure; CI, cardiac index; NYHA, New York Heart Association; 6MWD, 6-min walk distance; N/A, not applicable; UF, unifocalization; BT, Blalock–Taussig; RV–PA, right ventricle to pulmonary artery; 22q11.2, 22q11.2 deletion syndrome; IV, intravenous; INH, inhalation.

**Table 2:** Summary of Clinical Outcomes and Safety of PH Therapy in Patients with PA/VSD/MAPCA.

Outcome	Improved	Not Improved/Worsened	Not Reported
Clinical symptoms	19	2	2
Oxygen saturation (SpO_2_)	8	11	3
Pulmonary vascular resistance (PVR)	8	0	14
Pulmonary artery pressure (PAP)	7	2	13
Cardiac index	3	5	14
Adverse events	5	–	–
Treatment discontinuation due to adverse events	2	–	–
Mortality	4 (18%)	–	–

Numbers represent the number of patients. Percentages are shown for mortality only, reflecting the proportion of deaths among the total cohort (*n* = 22). Outcome-specific denominators vary due to heterogeneous reporting across studies.

## Data Availability

All data supporting the findings of this study are available within the paper and its Supplementary Information.

## Supplementary Materials

The following supporting information can be downloaded at: https://www.mdpi.com/article/10.3390/jcm15031087/s1, Table S1: PRISMA2020 checklist [21]; Table S2: Database Search Strategy and Results; Figure S1: Risk of bias in non-randomized studies of interventions according to the Cochrane ROBINS-I tool.

## Abbreviations

The following abbreviations are used in this manuscript:

PA	Pulmonary atresia
VSD	Ventricular septal defect
MAPCA	Major aortopulmonary collateral arteries
PH	Pulmonary hypertension
IPAH	Idiopathic pulmonary arterial hypertension
CHD	Congenital heart defect
RCT	Randomized controlled trial
PAP	Pulmonary artery pressure
SpO_2_	Peripheral oxygen saturation
PVR	Pulmonary capillary vascular resistance
