# Supplementary material for: Systematic Review of the Application of Pulmonary Hypertension Treatments in Ventricular Septal Defect, Pulmonary Atresia, and Major Aortopulmonary Collateral Arteries
Source: J Clin Med. 2026 Jan 30;15(3):1087. doi: 10.3390/jcm15031087 (PMC12898845; doi:10.3390/jcm15031087)

Figure S1. Risk of bias in non-randomized studies of interventions according to the Cochrane ROBINS-I tool

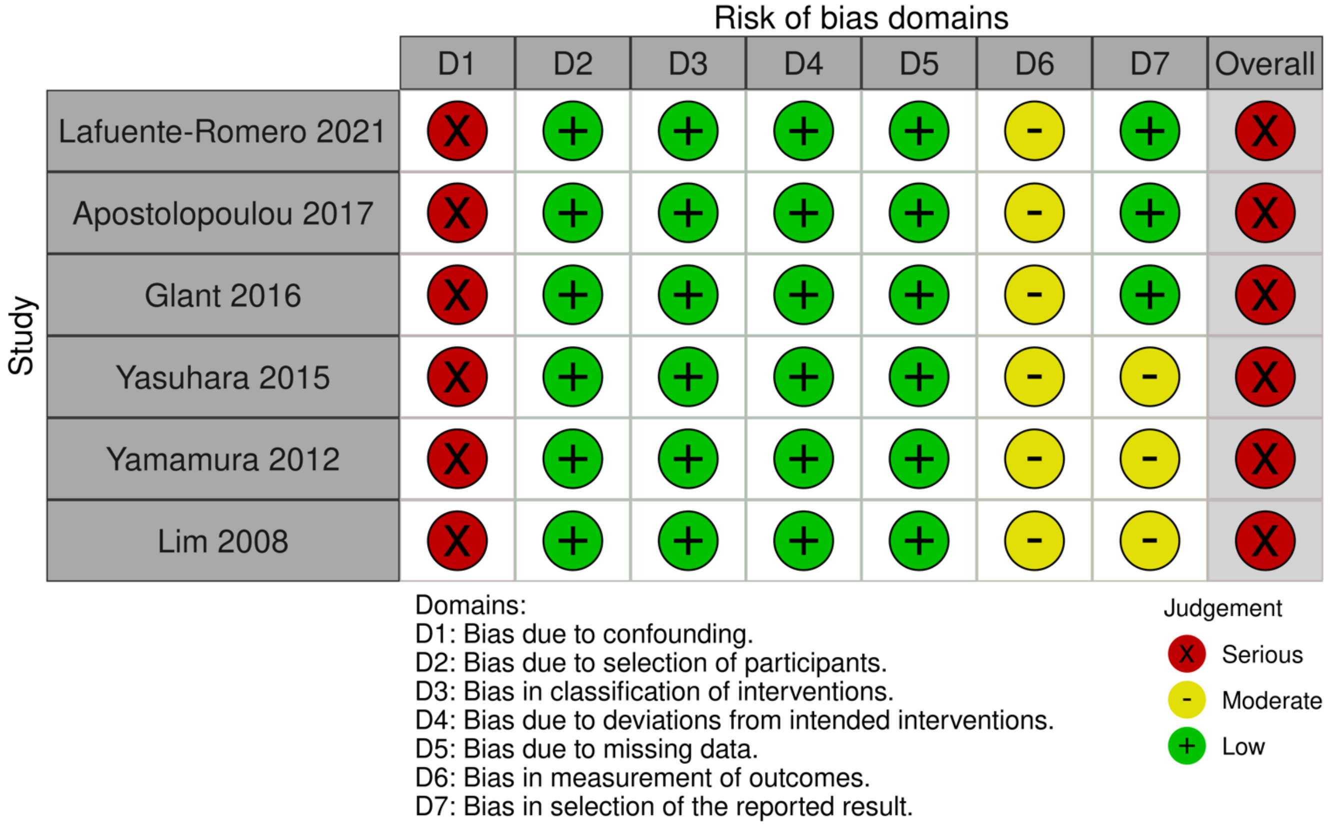

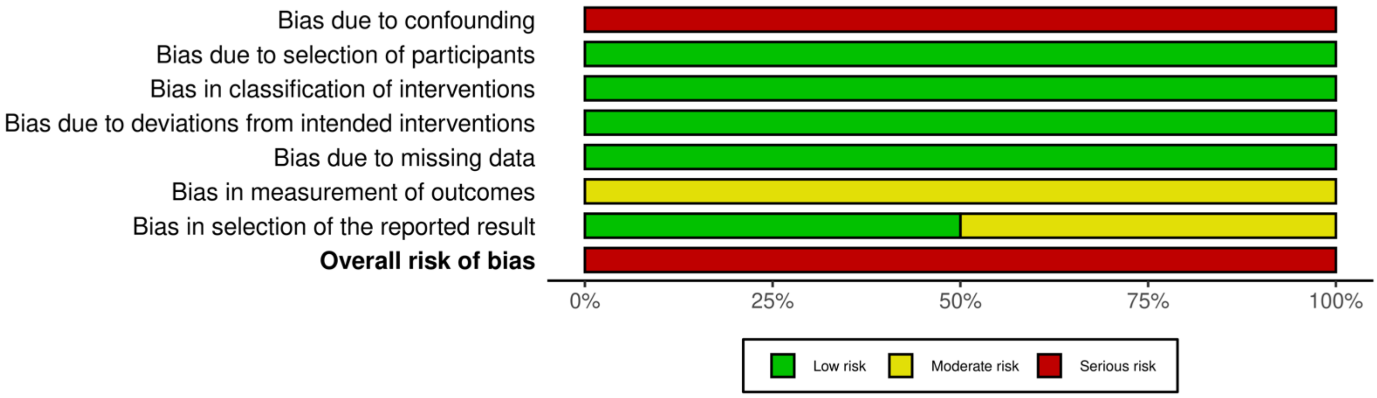

Supplement: Supplementary file 1 [file jcm-15-01087-s001.zip › Figure S1. ROBINS-I.pdf]
