# Supplementary material for: Systematic Review of the Application of Pulmonary Hypertension Treatments in Ventricular Septal Defect, Pulmonary Atresia, and Major Aortopulmonary Collateral Arteries
Source: J Clin Med. 2026 Jan 30;15(3):1087. doi: 10.3390/jcm15031087 (PMC12898845; doi:10.3390/jcm15031087)
Supplement: Supplementary file 1 [file jcm-15-01087-s001.zip › ●Table S2. Database Search Strategy and Results.pdf]

Table S2. Database Search Strategy and Results

Search strategies using Pubmed (Table S2a), MEDLINE (Table S2b), and Cochrane Cochrane Central Register of Controlled Trial (Table S2c) are shown below. Search using Igaku Chuo Zasshi (ICHUSHI) was performed in Japanese, and the search strategy will be provided upon request.

Table S2a. Search strategy using Pubmed

No.	Query	Hit retrieved
S1	(tetralogy of fallot) AND (major aortopulmonary collateral arteries) AND (pulmonary hypertension)	13
S2	(tetralogy of fallot) AND (aortopulmonary collateral artery) AND (pulmonary hypertension)	17
S3	(pulmonary atresia with ventricular septal defect) AND (major aortopulmonary collateral arteries) AND (pulmonary hypertension)	5
S4	(pulmonary atresia with ventricular septal defect) AND (aortopulmonary collateral artery) AND (pulmonary hypertension)	5
S5	S1 OR S2 OR S3 OR S4	19
S6	(treatment) OR (therapy)	13,160,014
S7	S5 AND S6	18

Table S2b. Search strategy using MEDLINE

No.	Query	Hit retrieved
S1	(tetralogy of fallot) AND (major aortopulmonary collateral arteries) AND (pulmonary hypertension)	64
S2	(tetralogy of fallot) AND (aortopulmonary collateral artery) AND (pulmonary hypertension)	21
S3	(pulmonary atresia with ventricular septal defect) AND (major aortopulmonary collateral arteries) AND (pulmonary hypertension)	34
S4	(pulmonary atresia with ventricular septal defect) AND (aortopulmonary collateral artery) AND (pulmonary hypertension)	12
S5	S1 OR S2 OR S3 OR S4	95
S6	(treatment) OR (therapy)	14,869,823
S7	S5 AND S6	68

Table S2c. Search strategy using Cochrane

No.	Query	Hit retrieved
S1	(tetralogy of fallot) AND (major aortopulmonary collateral arteries) AND (pulmonary hypertension)	0
S2	(tetralogy of fallot) AND (aortopulmonary collateral artery) AND (pulmonary hypertension)	0
S3	(pulmonary atresia with ventricular septal defect) AND (major aortopulmonary collateral arteries) AND (pulmonary hypertension)	0
S4	(pulmonary atresia with ventricular septal defect) AND (aortopulmonary collateral artery) AND (pulmonary hypertension)	0

S5	S1 OR S2 OR S3 OR S4	0
S6	(treatment) OR (therapy)	8,857
S7	S5 AND S6	0
